# The impact of India’s accredited social health activist (ASHA) program on the utilization of maternity services: a nationally representative longitudinal modelling study

**DOI:** 10.1186/s12960-019-0402-4

**Published:** 2019-08-19

**Authors:** Smisha Agarwal, Sian L. Curtis, Gustavo Angeles, Ilene S. Speizer, Kavita Singh, James C. Thomas

**Affiliations:** 1 Department of International Health, Johns Hopkins Bloomberg School of Public Health, W5009E, 615 N Wolfe Street, Baltimore, MD 21205 USA; 2 Maternal and Child Health, Gillings School of Global Public Health, University of North Carolina, Chapel Hill, NC USA; 3 Carolina Population Center, University of North Carolina, Chapel Hill, NC USA; 4 Department of Epidemiology, Gillings School of Global Public Health, University of North Carolina, Chapel Hill, NC USA

**Keywords:** Primary health care, Community health workers, India, Antenatal care, Maternity care, Impact evaluation, Accredited Social Health Activist

## Abstract

**Background:**

In 2006, the Government of India launched the accredited social health activist (ASHA) program, with the goal to connect marginalized communities to the health care system. We assessed the effect of the ASHA program on the utilization of maternity services.

**Methods:**

We used data from Indian Human Development Surveys done in 2004–2005 and in 2011–2012 to assess demographic and socioeconomic factors associated with the receipt of ASHA services, and used difference-in-difference analysis with cluster-level fixed effects to assess the effect of the program on the utilization of at least one antenatal care (ANC) visit, four or more ANC visits, skilled birth attendance (SBA), and giving birth at a health facility.

**Results:**

Substantial variations in the receipt of ASHA services were reported with 66% of women in northeastern states, 30% in high-focus states, and 16% of women in other states. In areas where active ASHA activity was reported, the poorest women, and women belonging to scheduled castes and other backward castes, had the highest odds of receiving ASHA services. Exposure to ASHA services was associated with a 17% (95% CI 11.8–22.1) increase in ANC-1, 5% increase in four or more ANC visits (95% CI − 1.6–11.1), 26% increase in SBA (95% CI 20–31.1), and 28% increase (95% CI 22.4–32.8) in facility births.

**Conclusions:**

Our results suggest that the ASHA program is successfully connecting marginalized communities to maternity health services. Given the potential of the ASHA in impacting service utilization, we emphasize the need to strengthen strategies to recruit, train, incentivize, and retain ASHAs.

## Introduction

In 2015, India accounted for approximately 45 000 (range 36 000 to 56 000) pregnancy-related maternal deaths, making it one of two countries that account for one third of all maternal deaths globally [1]. Between 1990 and 2016, the maternal mortality ratio (measured as a pregnancy-related deaths per 100 000 live births) declined by 77% [1]. While some progress was made to achieve the Millennium Development Goal of a two-third reduction in maternal mortality, the target was not met and progress varied substantially across states [2]. Between 2005–2006 and 2015–2016, the coverage of four or more antenatal care visits (ANC) increased from 37 to 51%, institutional deliveries increased from 39 to 79%, and percentage of births with a skilled attendant increased from 47 to 81% [3].

A number of studies have demonstrated the positive impact of community health worker (CHW) programs on the promotion of reproductive health services and family planning, appropriate care seeking, antenatal care during pregnancy, and skilled care for childbirth [4–8]. However, there have also been several concerns about the performance and accountability of CHW programs, especially in programs scaled beyond the efficacy settings [9]. To date, most of the studies on the effectiveness of CHW programs have been small-scale randomized trials with interventions delivered under controlled settings; limited evidence exists on the effectiveness of national-level CHW programs. The WHO guidelines on health policy and system support to optimize CHW programs highlights the need for using longitudinal methods to assess the long-term impact of CHW programs [10]. India’s accredited social health activist (ASHA) program is the largest government-led CHW program globally with nearly one million trained CHWs.

In response to the relatively slow progress to strengthen maternal and child health, the Government of India (GoI) launched the National Rural Health Mission (NRHM) in April 2005. The NRHM is an ambitious effort to strengthen the national health systems and health care delivery, with a special focus on improving health care outcomes among the poorest populations [11]. The ASHA program, considered vital to the success of the NRHM, aims to increase community engagement with the health system and support access to public health services [12, 13]. The ASHA program was first launched in 2006 in 18 high-focus states—including 10 high-focus states (Bihar, Jharkhand, Madhya Pradesh, Chhattisgarh, Himachal Pradesh, Jammu and Kashmir, Uttar Pradesh, Uttaranchal, Orissa, and Rajasthan) and 8 northeastern high-focus states (Arunachal Pradesh, Manipur, Assam, Nagaland, Meghlaya, Tripura, Mizoram, and Sikkim). Within 2 years, over 300 000 ASHAs were trained and deployed at a ratio of one ASHA to 1000 population in rural areas. In 2009, the program was expanded to the rest of the country [14]. As of December 2015, there were 937 595 ASHAs operating nationally. Of these, 544 074 ASHAs are in high-focus states, 56 104 in the northeastern states, and 337 417 in other non-high-focus states [15, 16]. The ASHAs are an all-female cadre of CHWs, selected based on national guidelines by local village committees. Though they are considered volunteers, they receive some performance-based incentives. Although variations exist across states in the recruitment, training, responsibilities, incentives, and supervision systems for the ASHAs, there are some common guiding tenets. Table 1 summarizes their training [17], responsibilities specific to promoting the health of mothers and children [14, 18], and compensation structure [19, 20].

**Table 1:** Training and responsibilities of ASHAs to promote the health of mothers and children

Recruitment - ASHAs must be female, between 21 and 45 years old with middle-school education (class eight or higher), and ideally should be a “daughter-in-law” of the village, either married, widowed, or divorced with a likelihood to live in the village for the foreseeable future. - States have flexibility in selecting ASHAs with lower literacy levels to ensure adequate community representation and local residence Training - The ASHAs receive training supported by the Government of India for 23 days spread over 12 months. Training models may vary by state and may involve partnerships with various NGOs and other training centers. - The ASHAs are expected to attend periodic review meetings and ongoing job training (12 additional days per year). Primary responsibilities - Expected to work about 2.3 h per day and 4 days per week, except during events such as training and immunization days. - Create awareness and provide information to the community on the determinants of health such as nutrition, basic sanitation and hygiene, and existing health services. - Counsel mothers on birth preparedness, safe delivery, feeding practices, immunization, prevention of common infections, and family planning. - Registering all pregnant women, provide three antenatal visits and two postnatal visits, and facilitate access to health services for the mother and child. - Rollout of other government programs such as the *Janani Surakshna Yojana* (JSY)—a cash entitlement program to incentivize women to give birth in health facilities. - Arrange escort or accompany pregnant women and children requiring treatment to health facilities. - Additional responsibilities of the ASHAs may vary by state. - Act as a bridge between the rural population and the government health system. Compensation - The ASHAs are honorary volunteers and receive performance-based compensation based on reported activities. - The compensation varies by the state and by the type of services provided. It ranges from INR 200 (~ $2.95) for registering a pregnant woman, providing 3 antenatal and 2 postnatal visits to INR 200–350 (~ $2.95–5) for facilitating institutional birth. Supervision - As per national guidelines, one ASHA facilitator is assigned for every 20 ASHAs, to help with selection, provide on-the-job mentoring, conduct regular supervisory meetings, and maintain records. - During monthly performance monitoring meetings, ASHAs are to report on their activities, especially around critical tasks around visiting newborns on the first day for home deliveries, attending immunization camps, visiting households to discuss nutrition, and acting as DOT providers for tuberculosis treatment.	

Given the size and scope of the ASHA program, an assessment of the program has relevance not only for the GoI, but also for other countries investing in similar CHW programs. Over the last decade, the ASHA program has been scaled nationally, with a budget allocation ranging from 80 million rupees (~ $1.2 million) to 1.05 billion rupees (~ $15.5 million) per state, between 2005 and 2010 alone [14, 21]. Furthermore, governments in other South Asian and African countries such as Ethiopia, Bangladesh, Kenya, Uganda, Ghana, and South Africa are in various stages of training and deploying cadres of CHWs to fill critical gaps in delivering health services to women and children [22]. Despite the importance of such programs, the evidence on their effectiveness, especially at scale, is limited.

Previous assessments of the effect of the ASHA program on maternity care have been descriptive [23], limited to certain states [14], or limited to assessing the performance of the ASHAs [11, 24] and not the impact of the ASHA program on health care outcomes. In this study, we investigate whether the ASHA program is reaching its target populations and assess whether the receipt of ASHA services is associated with an increase in the utilization of maternity care services.

## Methods

We used data from two rounds of the nationally representative, longitudinal Indian Human Development Surveys (IHDS). The surveys were funded by the National Institutes of Child Health and Human Development (NICHD) and were produced jointly by the National Council of Applied Economic Research (NCAER), New Delhi, and the University of Maryland. IHDS-I (2004–2005) was administered to 41 554 households—27 010 rural and 13 126 urban households. The rural sample was drawn using stratified random sampling of defined units. In urban areas, a stratified sample of towns and cities within states was selected by probability proportional to population size [25, 26]. Eighty-three percent of the households interviewed in 2004–2005, were re-interviewed in IHDS-II (2011–2012), and an additional replacement sample of 2134 households was added. Appendix 3 presents an analysis of households that were not interviewed in IHDS-II and were lost to follow-up. The IHDS-II survey was administered to 42,152 households in 33 states and union territories, 384 districts (of 612 districts), 1503 villages, and 971 urban areas.

Both rounds of the IHDS survey include a household interview with information about household asset ownership, and an interview with ever-married women of reproductive ages (15–49 years) with information about birth history, reproductive health, and antenatal and delivery care for the most recent birth. IHDS-I recorded 11 670 births, and IHDS-II recorded 13 881 births (demographic characteristics presented in Appendix 1). The implementation of the ASHA program started in April 2005. IHDS-I covers births from 2000 to 2005, and IHDS-II covers a 6-year period (2005–2011) after the implementation of the ASHA program.

### Measurement of program exposure and outcomes

We estimated the effect of the ASHA program on four outcomes: (1) whether the respondent received at least one ANC visit, (2) had 4 or more ANC visits, (3) delivered in a health facility, and (4) had a skilled attendant present at the time of birth. Following the 2008 WHO recommendations, we considered medical doctors, nurses, and auxiliary nurse midwives (ANMs) as skilled birth attendants [27].

IHDS-II survey gathered information on the type of health care provider seen by women at any point during their last pregnancy. A woman was considered exposed to an ASHA if she reported that an ASHA assisted her in response to at least one of the following questions: “Where did you get a pregnancy card made?”; “Did you get help from anyone for making a pregnancy card/registration?”; “Who visited you when you were pregnant?”; “Who facilitated or motivated you to go to a health facility for delivery?”; and “Who arranged the transportation to take you to the health facility for delivery?”. We explored three specifications for defining the exposure: (S1) Cluster-level “intensity” of ASHA exposure was calculated as number of women who reported exposure to an ASHA in a cluster during the last birth divided by the total number of eligible women who had a birth in 6 years preceding the survey in the cluster. This measure takes a value between 0 and 1 and captures direct individual exposure to an ASHA, as well as indirect effect that may result from the presence of an ASHA within the community. (S2) All women in clusters in which at least one woman reported seeing an ASHA received a value of “exposed” (i.e., 1). (S3) Women received a value of either 0 or 1 depending on their individual reported exposure.

### Characteristics of the women who report receipt of ASHA services

To understand whether the ASHA program is reaching its target population, we calculated the uptake of the program nationally, disaggregated by demographic characteristics, based on self-reported ASHA exposure (S3) using IHDS-II data. To understand whether ASHAs are differentially used by individuals belonging to a certain demographic in areas where the ASHA program is active, we restricted the analysis to only those clusters where ASHA services were reported. We used logistic regression to investigate the association between a range of individual and household characteristics and receipt of ASHA services in clusters where an ASHA has been reported. We estimated this model at the national level and separately for rural areas and high-focus states.

The logistic models account for sociodemographic characteristics of the women—including maternal age (15–19, 20–24, 25–29, 30–34, 35–39, 40–49 years), maternal education (1–5 years, secondary 6–11 years, 12 years or more of education), maternal caste (upper/forward caste, scheduled caste (SC), scheduled tribe (ST), other backward caste (OBC)), religion (Hindu, Muslim, other religions), parity, and household wealth index. OBC, SC, and ST are official Government of India caste classifications for minority groups of historically disadvantaged people. Continued disparities by caste exist in education, income, and social networks. We used polychoric principal component analysis (PCA) to estimate household wealth using information about household asset ownership (bicycle, sewing machine, generator, mixer, motorcycle, television, air cooler, clock, fan, chair/table, cot, telephone, mobile phone) and household characteristics (type of cooking place, type of toilet, availability of electricity, type of *chulha* (hearth), water source, wall type, roof type, floor type) from the IHDS household surveys. The polychoric procedure, unlike the standard PCA, retains ordinal variables without breaking them into dummy variables [28]. The first component of the polychoric PCA was used to create wealth quintiles, explaining 27% of the variance for the 2005 and 30% for the 2012 data.

### Association between exposure to ASHAs and maternal service utilization outcomes

We assessed the effect of exposure to ASHAs, measured as cluster-level exposure intensity (S1), on the outcomes, using multivariate difference-in-differences models fitted using ordinary least squares regression [29]. The difference-in-differences approach assesses the effect of the ASHA program by controlling for baseline differences between exposed and unexposed populations, and for temporal differences that may have resulted from underlying changes over time [30]. We used fixed effects at the cluster level to control for baseline differences between clusters and any unmeasured time-invariant factors that may have resulted in selective uptake or targeting of the ASHA program. The models controlled for individual and household characteristics: maternal age, maternal education, household wealth index, birth order, maternal caste, and religion. All regression models were adjusted for survey design features using cluster-level sample weights, and standard errors were corrected for correlation across individuals in the same cluster using robust standard errors.

We tested the sensitivity of our findings to various model specifications. We estimated the models separately for rural areas and for high-focus states (results not presented). About 30% of the clusters had 3 or fewer eligible women. Since ASHA exposure is defined as the cluster average, we tested the robustness of the results to cluster sizes greater than two and greater than three. Additionally, we also used the three different specifications for exposure to the ASHA program for further robustness checks. Around 2005, a national cash assistance program called *Janani Surakshna Yojana* was launched to incentivize women living below the poverty line to deliver in health facilities or with a skilled attendant [31]. The ASHAs play a vital role in rolling out this program. We could not control for cash assistance in the above models for skilled birth attendance (SBA) and facility birth, as data on cash transfer were only available for women who delivered in a health facility. To disentangle the effect of the cash transfer on SBA and facility births from the effect of the ASHA program alone, we tested a multinomial logistic model with SBA and facility birth coded as three-level categorical outcomes. Facility births were coded as birth at home, birth in a health facility without any financial incentive, and birth in a health facility with a financial incentive, and SBA was categorized as no SBA, SBA without financial incentive, and SBA with financial incentive.

## Results

**Fig. 1:**
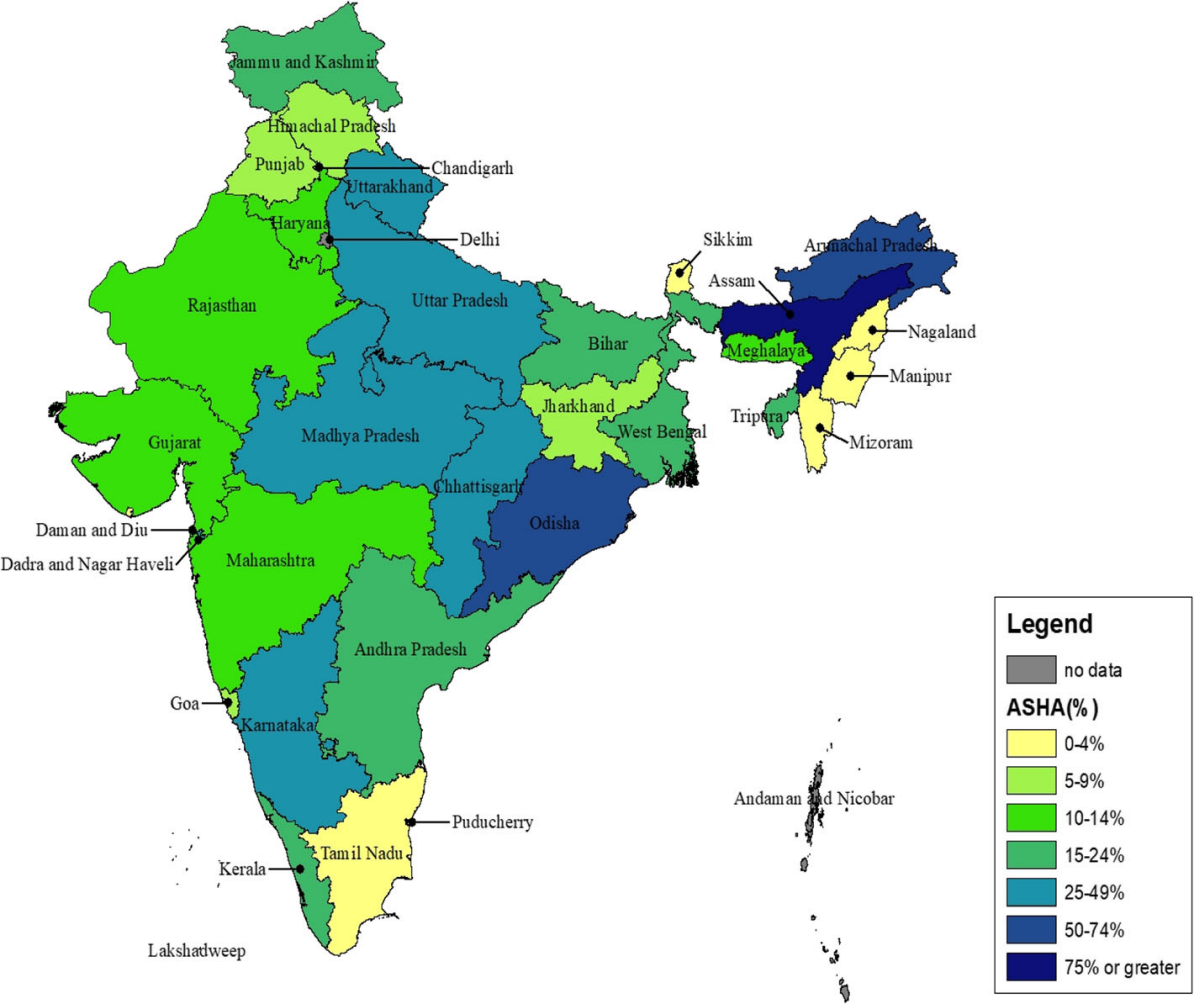
Percentage of women reporting receiving services from an ASHA among all women who had a live birth since 2005

**Fig. 2:**
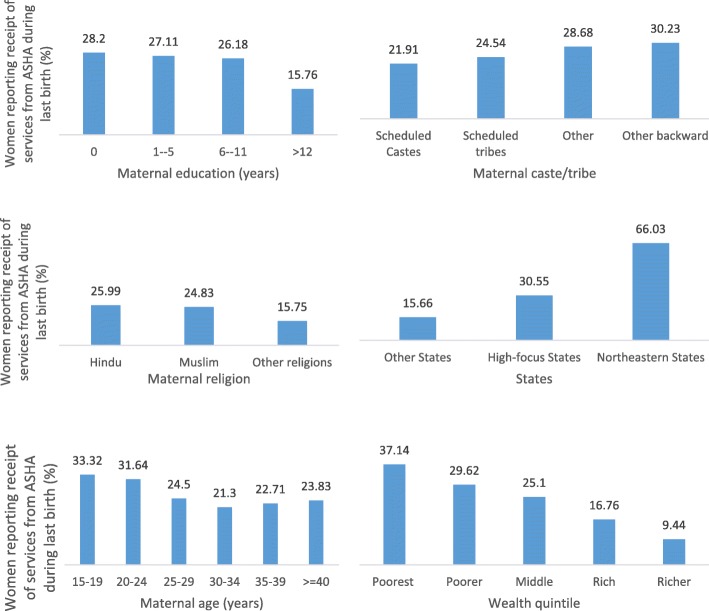
Percentage of women reporting receipt of ASHA services among all women who gave birth from 2005 to 2011

Nationally, 25% of women who had a live birth since 2005 reported receiving one or more ASHA services during their last pregnancy (measured as proportion of women who reported receiving ASHA services during their last pregnancy, among all women who had a live birth since 2005). Fifty-nine percent of women interviewed in IHDS-II live in clusters where an ASHA has been reported. Substantial variations in the receipt of ASHA services were reported at the state level (Fig. 1). Sixty-six percent of women in northeastern states, 30% of women from high-focus states, and 16% of women in other states reported receipt of ASHA services (Fig. 2). Over 30% of all rural women reported ASHA services, compared to less than 10% of urban women.

To understand whether ASHAs were differentially targeting any demographic groups within clusters where they are active, we restricted the analysis to only those 59% (*n* = 7985) of the clusters that reported an ASHA (Table 2). Compared to the youngest women ages 15–19 years, women in age groups 30–34, 35–39, and > 40 were significantly less likely to report ASHA services in the restricted sample. Within clusters with an active ASHA, compared to women with no education, women with 6–11 years and > 12 years of education were more likely to report receipt of ASHA services. This pattern was observed nationally, as well as when the sample is restricted to rural areas and to high-focus states. Compared to forward castes, women belonging to disadvantaged groups of OBC and scheduled castes are more likely to report ASHA services. However, no significant difference in receipt of ASHA services was observed among women belonging to scheduled tribes. Within clusters with an active ASHA, odds of reporting ASHA services declined steadily with increasing wealth status—nationally, in rural areas, and in high-focus states. No significant differences in the receipt of ASHA services were reported by religion across all groups.

**Table 2:** Analysis of association between receipt of ASHA services for the most recent birth and individual characteristics using logistic regression and the 2011–2012 IHDS survey

	Restricted clusters* (*n* = 7 985)			Rural areas only (*n* = 6 854)			High-focus states (*n* = 4 662)		
	Odds ratio(95% CI)		*p* value	Odds ratio(95% CI)		*p* value	Odds ratio(95% CI)		*p* value
Maternal age (years)									
15–19	1			1			1		
20–24	1.04	(0.74–1.48)	0.8057	1.13	(0.78–1.63)	0.5186	0.99	(0.62–1.58)	0.9628
25–29	0.80	(0.57–1.13)	0.2047	0.87	(0.60–1.26)	0.4534	0.83	(0.52–1.33)	0.4485
30–34	0.63	(0.44–0.91)	0.0131	0.65	(0.44–0.97)	0.0339	0.62	(0.38–1.04)	0.0683
35–39	0.64	(0.43–0.95)	0.0265	0.69	(0.46–1.06)	0.0893	0.61	(0.36–1.04)	0.0709
≥ 40	0.63	(0.41–0.99)	0.0461	0.70	(0.43–1.14)	0.1505	0.58	(0.32–1.04)	0.0665
Education				–					
No education	1			1			1		
1–5 years	1.13	(0.97–1.31)	0.1178	1.11	(0.94–1.30)	0.2122	1.12	(0.94–1.35)	0.2114
6–11 years	1.44	(1.26–1.66)	0.0000	1.53	(1.32–1.78)	0.0000	1.46	(1.23–1.73)	0.0000
12 years or more	1.33	(1.09–1.63)	0.0055	1.4	(1.09–1.72)	0.0068	1.30	(0.97–1.73)	0.0764
Caste									
Forward caste	1			1			1		
Backward castes	1.17	(0.99–1.37)	0.0638	1.16	(0.96–1.40)	0.1152	1.34	(1.09–1.65)	0.0050
Scheduled castes	1.30	(1.08–1.57)	0.0053	1.25	(1.02–1.54)	0.0355	1.59	(1.25–2.02)	0.0002
Scheduled tribes	1.19	(0.92–1.54)	0.1843	1.17	(0.89–1.55)	0.2629	1.30	(0.92–1.82)	0.1369
Religion									
Hindu	1			1			1		
Muslim	1.05	(0.85–1.30)	0.6579	1.06	(0.82–1.37)	0.6529	0.96	(0.72–1.28)	0.7711
Other religions	0.79	(0.57–1.08)	0.1392	0.81	(0.56–1.16)	0.2414	1.55	(0.62–3.91)	0.3534
States									
Other states	1			1			1		
High-focus states	1.22	(1.04–1.43)	0.0149	1.27	(1.06–1.52)	0.0095			
Northeastern states	5.94	(3.80–9.29)	0.0000	6.73	(3.95–11.46)	0.0000			
Urban/rural residence									
Urban	0.90	(0.75–1.08)	0.2630				0.88	(0.66–1.16)	0.3545
Rural	1						1		
Wealth quintile									
Lowest	1			1			1		
Lower	0.76	(0.66–0.88)	0.0002	0.75	(0.64–0.87)	0.0001	0.76	(0.64–0.90)	0.0015
Middle	0.78	(0.66–0.92)	0.0040	0.80	(0.67–0.96)	0.0150	0.81	(0.65–1.01)	0.0591
Richer	0.55	(0.45–0.67)	0.0000	0.51	(0.41–0.63)	0.0000	0.53	(0.41–0.70)	0.0000
Richest	0.39	(0.31–0.49)	0.0000	0.35	(0.26–0.45)	0.0000	0.36	(0.26–0.51)	0.0000
Parity	0.93	(0.89–0.97)	0.0006	0.93	(0.89–0.97)	0.0008	0.95	(0.91–1.00)	0.0524

*Analysis is restricted only to clusters where at least one woman reported seeing an ASHA

Between 2005 and 2012, the use of at least one antenatal care increased from 74 to 84%, the use of a skilled attendant at birth increased from 53 to 75%, and the use of health facilities for giving birth increased from 43 to 66%. Table 3 presents three models to assess the relationship between exposure to ASHAs (exposure S1) and utilization of maternity services using a difference-in -differences model—a crude model (M1) without any confounders, a model (M2) with confounding covariates, and a model (M3) with confounding covariates and cluster-level fixed effects. Across all four outcomes, the crude model suggests that exposure to ASHAs is associated with a decrease in the use of the maternity services. However, this relationship is reversed, and a positive association is seen when the confounding variables are accounted for in M2; the positive relationship is further strengthened when cluster-level fixed effects are accounted for in M3. The reversal of the association may be explained by the results in Table 2 that show that receipt of ASHA services is more likely to be reported by the most marginalized women—women who are also least likely to have care-seeking behaviors.

**Table 3:** Association between intensity of exposure to the ASHA program and maternity service utilization outcomes using a linear probability model

	M1 (crude model)			M2 (M1 + other covariates)			M3 (M2 + cluster fixed effects)		
	*N*	ME*	(95% CI)	*N*	M	(95% CI)	*N*	ME	(95% CI)
Antenatal care, one visit	24 763	− 0.032	(− 0.067–0.003)	24 565	0.12	(0.094–0.156)**	24 565	0.17	(0.118–0.221)**
Antenatal care, four visits	24 777	− 0.215	(− 0.269–0.162)*	24 578	0.038	(0.011–0.088)	24 578	0.047	(− 0.016–0.111)
Skilled birth attendance	24 613	− 0.047	(− 0.091–0.003)*	24 420	0.200	(0.163–0.237)**	24 240	0.258	(0.206–0.310)**
Birth at a health facility	24 721	− 0.029	(− 0.077–0.020)	24 519	0.245	(0.204–0.286)**	24 519	0.276	(0.224–0.328)**
Estimated marginal effects (ME) are the change in predicted probabilities (95% CI) as a result of receipt of ASHA services, controlling for maternal age, maternal education, caste, religion, parity, socioeconomic status, and rurality *p < 0.05; **p < 0.01									

**Fig. 3:**
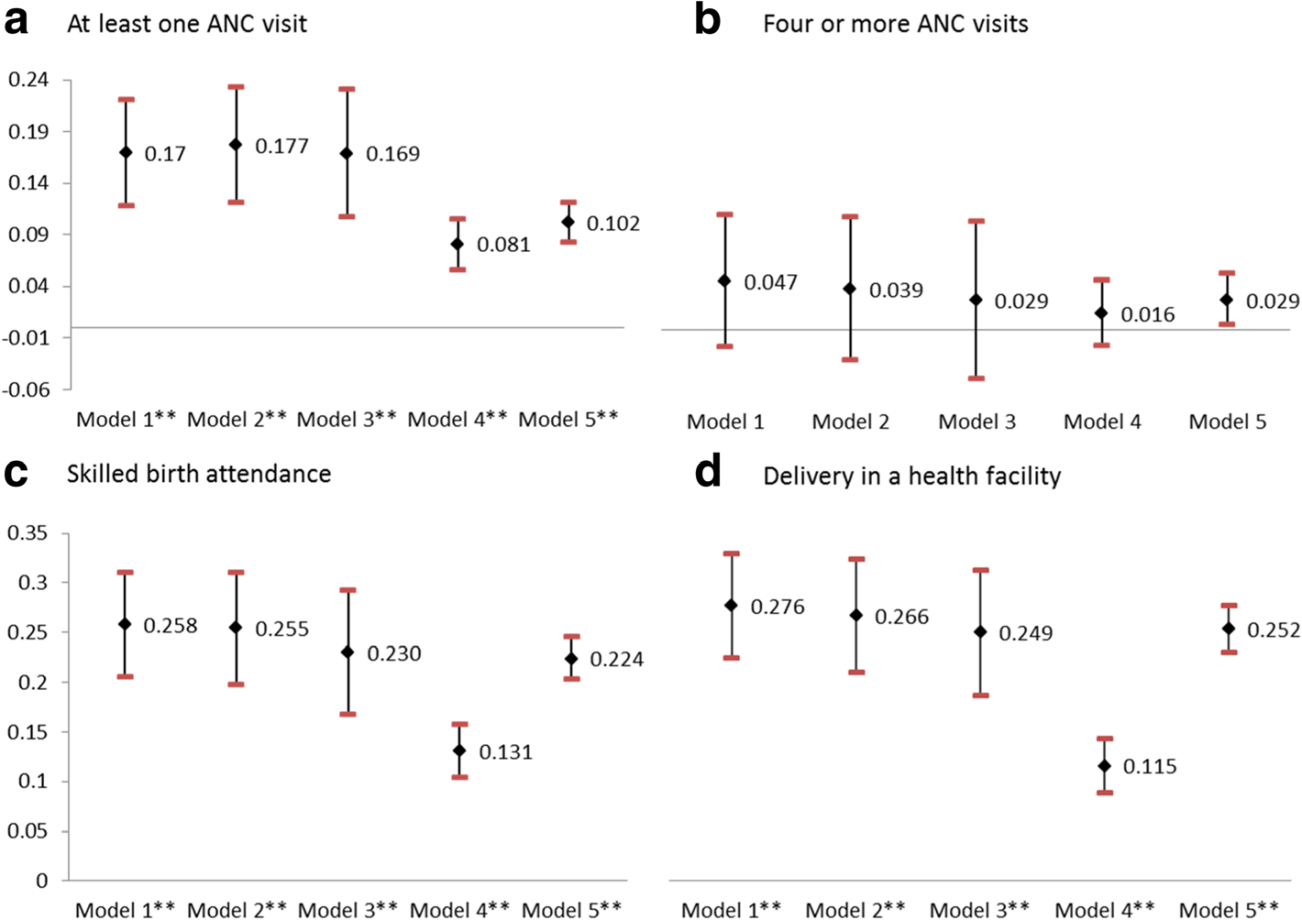
Sensitivity analysis for association between exposure to ASHA and utilization of maternity services using a linear probability model. **p < 0.05; **p < 0.01. For each outcome, the point estimates and 95% confidence intervals (indicated by the whiskers) correspond to program effect sizes estimated using difference-in-differences models fitted using ordinary least squares regression, controlling for a range of potentially confounding variables. Model 1: S1 exposure definition (cluster-level intensity of ASHA exposure measured as the number of women who reported exposure to an ASHA in a cluster during the last birth divided by the total number of eligible women who had a birth in 6 years preceding the survey in the cluster); Model 2: S1, for clusters > 2; Model 3: S1, for clusters > 3; Model 4: S3 exposure definition (Exposure is coded based on individual women’s response to whether they received ASHA services—0 for No, 1 for Yes); Model 5: S2 exposure definition (All women in clusters in which at least one woman reports seeing an ASHA received a value of “exposed” (i.e., 1)). All models control for cluster-level fixed effects

Exposure to ASHAs is associated with a 17% increase in the utilization of at least one antenatal care visit, a 5% increase in the utilization of 4 or more antenatal care visits, a 26% increase in SBA, and a 28% increase in giving birth in a health facility (Table 3). This implies that for every 10 women receiving ASHA services, one or two additional women would receive at least one ANC visit, and additional two or three women would give birth in a health facility or with a skilled birth attendant outside a facility. High maternal education and household wealth, upper castes, and lower parity were associated with increased probability of having ANC, SBA, and giving birth in a health facility (results presented in Appendix 2). The association between exposure to ASHAs and maternity outcomes was robust to variations in cluster size and exposure specifications. While the magnitude of the association varies for the different exposure specifications, the results still suggest a significant association between receipt of ASHA services and the outcomes (Fig. 3)

.

**Fig. 4:**
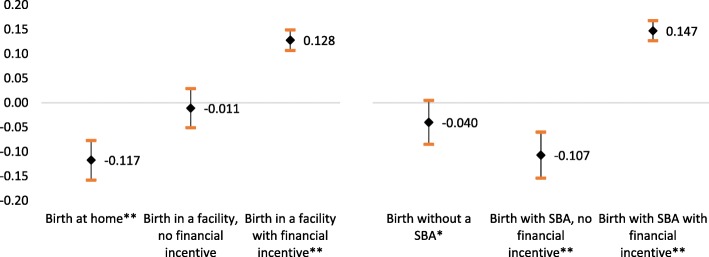
Marginal effect of ASHA exposure on the facility deliveries and skilled birth attendance, using a multinomial logistic model. For each outcome, the point estimates and 95% confidence intervals (indicated by the whiskers) correspond to program effect sizes estimated using a multinomial regression model, adjusted for a range of potentially confounding variables.

We attempted to understand the role of conditional cash transfers in motivating women to go to health facilities for birth and have a skilled attendant at the time of birth. Exposure to ASHAs is significantly associated with a reduction in home births, and births without a skilled attendant (Fig. 4). Exposure to ASHAs with financial incentives was significantly associated with a 12% and 15% increase in heath facility births and skilled birth attendance, respectively.

## Discussion

Exposure to ASHAs was strongly correlated with the utilization of maternity services. Even the most conservative assumptions for specifying exposure to ASHAs suggest that the ASHA program is associated with improved utilization of at least one antenatal care visit, skilled birth attendance, and giving birth in a health facility. The results also highlight the role of conditional cash transfers to incentivize facility-based births, and suggest that the presence of the ASHAs further augments this effect. The intent of the ASHA program was to reach marginalized communities—to connect communities that do not typically avail of services in health facilities to health care services at the community level. Our results suggest that the ASHA program is meeting these objectives as few programs have successfully done in the past.

While the ASHA program had the highest reach in the poorest populations, it does not address the disparities in the utilization of services across women from different socioeconomic and caste groups, especially the scheduled tribes. Historically, women belonging to scheduled tribes have minimal utilization of health services. For example, the National Family Health Survey (NFHS) reports facility-based delivery at 18% among scheduled tribes, compared to 33% among scheduled castes, and 51% among forward castes [32]. Our study indicates that utilization of maternity services continues to be the lowest among scheduled tribes, even after accounting for receipt of ASHA services. The tribal nature of these communities makes linkages to the health care system challenging and warrants further programming that is responsive to tribal customs around childbirth.

Prior process evaluations of the ASHA program highlighted several operational challenges. In several areas, the ASHAs do not receive timely payments for their services, which affects their performance [19]. Performance-based payments alone do not provide the financial security that is expected [33]. Studies conducted at the district or state level have reported that ASHAs may not be motivated in their role due to poor financial compensation [24, 34, 35]. Others have reported that ASHA activities in some states are hindered due to poor supportive supervision and mentoring structures [36]. Given our findings about the potential impact of the ASHA program on the utilization of services, especially for the marginalized, it is even more vital that these operational challenges are better understood and addressed. First, for the ASHA program to be sustainable, recruiting and training new ASHAs is as important as continued investments to ensure that the existing ASHAs have the institutional support and ongoing education needed to deliver the ever-expanding set of services expected of them. The effectiveness of large-scale mobile programs such as Mobile Academy for training of ASHAs should be robustly evaluated and, if effective, should serve as an adjunct instead of replacement for face-to-face instruction. Second, further research is needed to understand how the different implementation approaches, incentive systems, and support structures adopted by individual states have influenced the uptake and functioning of the ASHA program. Third, the role of the ASHAs in facilitating respectful maternity care needs to be further explored.

Contrary to the results of this study, a recent ecological analysis that explored the relationship between the change in proportion of villages within a district with an ASHA and a number of maternal health utilization outcomes, showed no impact of ASHA exposure on institutional delivery [37]. This study has several analytic improvements over the previous study in accounting for demographic characteristics which are established confounders of this relationship in the models, individual-level (instead of district-level) measurement of ASHA exposure and outcomes, and a longitudinal study design that accounts for targeting of the program by employing fixed effects methods at the cluster level. We assumed that accounting for cluster-level effects addresses variations in available health infrastructure thought of as critical to the success of the ASHAs. We used whether a woman received ASHA services as a proxy for whether the ASHA program was active in the area. By measuring ASHA exposure based on women’s self-reports, we capture functional exposure to the ASHA program. While this leaves room for measurement error in estimating the level of exposure, we account for this by presenting results from sensitivity analyses assuming a range of exposure definitions. Our study has some limitations. Seventeen percent of the households that were lost to follow-up in the IHDS-II survey were mostly urban, overall better-off households with higher levels of service utilization compared to the households that were part of both IHDS surveys. This limits the external validity of our findings. As with any non-experimental evaluation, our analysis is limited by unobserved factors associated with selective uptake of the ASHA program. Lastly, we are unable to disentangle the impact of the ASHAs from the impact of conditional cash transfers in driving the increase in SBA and facility births. Given that in most places the ASHAs bear the responsibility of informing women about the government’s conditional cash transfer scheme, we expect the impact of the two programs to be synergistic.

Our analysis presents an encouraging picture of the ASHA program 6–7 years into its implementation at scale. The ASHA program has increased in the utilization of antenatal care services, skilled birth attendance, and facility deliveries across caste, religion, and demographic groups. Most importantly, our analysis suggests that the ASHAs are more likely to reach groups that are typically left out of the formal health care system—poorer populations living in rural areas and women belonging to backward castes. The results of our study are promising given the sustained investments into the ASHA program by the national and state governments. However, the government needs to invest into supporting and increasing the reach of the ASHAs in marginalized regions with scheduled tribes. We emphasize the need to conduct ongoing monitoring and evaluation of the program to understand approaches to effectively scale the program and strengthen strategies to recruit, train, incentivize, and retain ASHAs.

In 2013, an expert consultation on the theme of CHWs in health care, organized by the United Nations Health Agencies (H4+) (UNAIDS, UNFPA, UNICEF, UN Women, the WHO, and the World Bank), highlighted the importance of CHW programs in strengthening national health systems and the critical need for identifying evidence-based interventions that CHWs can undertake in the reproductive health space. Identifying what interventions can be effectively delivered by CHWs would further strengthen the WHO and partner recommendations on task-sharing/task-shifting. This study addresses this gap by assessing the potential role of CHWs in effectively delivering maternity interventions and identifying the kind of interventions that are most likely to be affected through the engagement of CHWs. Historically, CHW programs at scale have not demonstrated the same impact as they have in controlled trials. Given the demonstrable impact of the ASHA program on the utilization of maternity services in our research, other countries and donor agencies can draw from the unique implementation features of the ASHA program in India.

## Appendix 1

**Table 4:** Demographic characteristics of the study sample in IHDS-I (2005) and IHDS-II (2012) surveys

	2005		2012	
	Total population		Total population	
	*N* = 11 670	%	*N* = 13 881	%
Maternal age (years)				
15–19	438	3.75%	294	2.12%
20–24	3 361	28.80%	3 180	22.91%
25–29	3 979	34.10%	5 051	36.39%
30–34	2 241	19.20%	3 197	23.03%
35–39	1 180	10.11%	1 455	10.48%
≥ 40	471	4.04%	704	5.07%
Education				
No education	5 312	45.52%	4 592	33.08%
1–5 years	1 774	15.20%	2 042	14.71%
6–11 years	3 287	28.17%	5 101	36.75%
12 years or more	1 091	9.35%	2 139	15.41%
Missing	204	1.75%	8	0.06%
Caste				
Forward caste	3 012	25.81%	3 512	25.30%
Other backward castes	4 953	42.44%	5 919	42.64%
Scheduled castes	2 706	23.19%	3 230	23.27%
Scheduled tribes	999	8.56%	1 191	8.58%
Missing			29	0.21%
Religion				
Hindu	9 413	80.66%	11 152	80.34%
Muslim	1 627	13.94%	2 121	15.28%
Others religions	630	5.40%	608	4.38%
States				
Other states	5 212	44.66%	6 081	43.81%
High-focus states	6 148	52.68%	7 278	52.43%
Northeast states	310	2.66%	522	3.76%
Urban/rural residence				
Rural	8 625	73.91%	9 886	71.22%
Urban	3 045	26.09%	3 995	28.78%
Wealth quintile				
Lowest	3 475	29.78%	3 766	27.13%
Lower	2 688	23.03%	2 918	21.02%
Middle	2 248	19.26%	2 553	18.39%
Richer	1 791	15.35%	2 440	17.58%
Richest	1 467	12.57%	2 204	15.88%

## Appendix 2

**Table 5:** Association between cluster-level exposure to ASHA measured as “intensity of exposure” and maternity service utilization outcomes using a linear probability model with fixed effects at the cluster level

	ANC-1 (*N* = 24 565)			ANC-4 (*N* = 24 578)			SBA (*N* = 24 240)			Facility birth (*N* = 24 519)		
	*β*(95% CI)		*p* value	*β*(95% CI)		*p* value	*β*(95% CI)		*p* value	*β*(95% CI)		*p* value
Outcome	0.17	(0.118–0.221)	0.000	0.047	(− 0.016–0.011)	0.146	0.258	(0.206–0.310)	0.000	0.276	0.224–0.326)	0
Maternal age (years)												
15–19	1			1			1			1		
20–24	− 0.012	(− 0.038–0.014)	0.362	0.012	(− 0.023–0.046)	0.503	0.003	(− 0.030–0.037)	0.848	− 0.001	(− 0.033–0.032	0.967
25–29	− 0.009	(− 0.034–0.018)	0.527	0.010	(− 0.026–0.045)	0.591	− 0.018	(− 0.051–0.015)	0.277	− 0.028	(− 0.061–0.005)	0.091
30–34	− 0.01	(− 0.039–0.019)	0.5	0.023	(− 0.013–0.060)	0.209	− 0.024	(− 0.059-0.010)	0.169	− 0.041	(− 0.076–0.006)	0.020
35–39	− 0.022	(− 0.053–0.009)	0.173	0.027	(− 0.012–0.067)	0.182	− 0.004	(− 0.042–0.034)	0.837	− 0.024	(− 0.062–0.014)	0.212
≥ 40	− 0.027	(− 0.067–0.013)	0.186	0.043	(− 0.002–0.088)	0.060	− 0.007	(− 0.053–0.038)	0.744	− 0.026	(− 0.071–0.019)	0.257
Education	–						–					
No education	1			1			1			1		
1–5 years	0.049	(0.034–0.065)	0.000	0.035	(0.017–0.053)	0.000	0.043	(0.024–0.062)	0.000	0.032	(0.013–0.051)	0.001
6–11 years	0.065	(0.052–0.079)	0.000	0.094	(0.076–0.112)	0.000	0.096	(0.079–0.113)	0.000	0.102	(0.085–0.118)	0.000
12 years or more	0.072	(0.056–0.088)	0.000	0.164	(0.139–0.188)	0.000	0.143	(0.123–0.164)	0.000	0.161	(0.139–0.183)	0.000
Caste	–											
Forward caste	1			1			1			1		
Other backward castes	− 0.017	(− 0.030–0.005)	0.008	− 0.009	(− 0.026–0.009)	0.344	− 0.020	(− 0.036––0.003)	0.017	− 0.026	(− 0.043–0.010)	0.002
Scheduled castes	− 0.019	(− 0.035–0.004)	0.015	− 0.036	(− 0.056–0.015)	0.001	− 0.028	(− 0.048–0.009)	0.004	− 0.04	(− 0.061–0.019)	0.000
Scheduled tribes	− 0.038	(− 0.064–0.013)	0.003	− 0.054	(− 0.086–0.021)	0.001	− 0.083	(− 0.115–0.051)	0.000	− 0.076	(− 0.107–0.044)	0.000
Religion	–											
Hindu	1			1			1			1		
Muslim	0.002	(− 0.018–0.023)	0.799	− 0.017	(− 0.042–0.008)	0.18	− 0.012	(− 0.036–0.012)	0.339	− 0.032	(− 0.057–0.006)	0.014
Others religions	− 0.004	(− 0.025–0.018)	0.75	0.021	(− 0.015–0.058)	0.254	− 0.022	(− 0.047–0.003)	0.083	− 0.019	(− 0.047–0.009)	0.181
Urban/rural residence	–											
Urban	− 0.038	(− 0.095–0.019)	0.188	− 0.054	(− 0.183–0.075)	0.413	− 0.082	(− 0.165–0.000)	0.051	− 0.054	(− 0.138–0.029)	0.200
Rural	1			1						1		
Wealth quintile												
Lowest	1			1			1			1		
Lower	0.053	(0.037–0.070)	0.000	0.038	(0.021–0.056)	0.000	0.053	(0.034–0.072)	0.000	0.024	(0.005–0.044)	0.014
Middle	0.078	(0.060–0.967)	0.000	0.071	(0.049–0.093)	0.000	0.089	(0.067–0.111)	0.000	0.067	(0.044–0.089)	0.000
Richer	0.092	(0.072–0.112)	0.000	0.094	(0.069–0.119)	0.000	0.123	(0.099–0.247)	0.000	0.104	(0.080–0.129)	0.000
Richest	0.109	(0.088–0.131)	0.000	0.157	(0.127–0.187)	0.000	0.164	(0.137–0.190)	0.000	0.154	(0.126–0.182)	0.000
Parity	− 0.022	(− 0.026–0.018)	0.000	− 0.02	(− 0.025–0.017)	0.000	− 0.031	(− 0.036–0.026)	0.000	− 0.031	(− 0.036–0.026)	0.000

## Appendix 3: Analysis of households that were lost to follow-up in IHDS-II

**Table 6:** Characteristics of households that were lost to follow-up in IHDS-II compared to households that were interviewed in both rounds

	Households lost to follow-up	Households interviewed in both rounds	*p* value
	(*N* = 6 911)	(*N* = 34 643)	
Avg. number of individuals in the household	4.24	5.38	0.000
Avg. number of children in the household	1.22	1.73	0.000
Avg. number of married women in the household	1.01	1.26	0.000
Avg. number of married men in the household	1.00	1.21	0.000
Avg. household asset score (0–30)	14.34	11.84	0.000
Caste/religion categories *N*(%)			0.000
Brahmin	564(8.16)	1 857(5.36)	
Upper caste	1 459(21.11)	5 692(16.43)	
Other backward caste	2 069(29.94)	11 999(34.64)	
Scheduled castes	1 081(15.64)	7 252(20.93)	
Scheduled tribes	530(7.67)	2 909(8.40)	
Muslims	904(13.08)	3 804(10.98)	
Other religions (Sikhs, Jains, Christians)	304(4.4)	1 130(3.26)	
Rural/urban *N*(%)			0.000
Rural	2 764(39.99)	23 970(69.19)	
Urban	4 147(60.01)	10 673(30.81)	

**Table 7:** Study outcomes among eligible women (with a live birth in the 5 years preceding the interviews) from households that were lost to follow-up in IHDS-II compared to women from households that were interviewed in both rounds

	Households lost to follow-up	Households interviewed in both rounds	*p* value
Any antenatal care			0.000
Total number	1 646	9 924	
No ANC	215(13.06)	2 247(22.64)	
At least one ANC visit	1431(86.94)	7 677(77.36)	
Skilled birth attendance			0.000
Total number	1 643	9 994	
Birth by non-medically trained provider	490(29.82)	4 457(45.09)	
Birth by medically trained provider	1 153(70.18)	5 427(54.91)	
Birth in a health facility			0.000
Total number	1 646	9 965	
Birth at home	580(35.24)	5 467(54.86)	
Birth in a health facility	1 061(64.46)	4 432(44.48)	

## Abbreviations

ANC: Antenatal care
ANM: Auxiliary nurse midwives
ASHA: Accredited social health activist
CHW: Community health worker
GoI: Government of India
IHDS: Indian Human Development Survey
MoHFW: Ministry of Health and Family Welfare
NCAER: National Council of Applied Economic Research
NFHS: National Family Health Survey
NICHD: National Institutes of Child Health and Human Development
NRHM: National Rural Health Mission
OBC: Other backward caste
PCA: Principal component analysis
PHC: Primary health care
SBA: Skilled birth attendance
SC: Scheduled caste
SDGs: Sustainable Development Goals
ST: Scheduled tribe
WHO: World Health Organization
